# A Case of Foix-Chavany-Marie Syndrome With Asynchronous Bilateral Opercular Infarcts and Chronic Bilateral Cerebellar Infarcts

**DOI:** 10.7759/cureus.26013

**Published:** 2022-06-16

**Authors:** Shreyan A Patel, Austin Forrester, Hyunwoong Kang, Eric Collin, Kashyap Patel

**Affiliations:** 1 Internal Medicine, Edward Via College of Osteopathic Medicine, Blacksburg, USA; 2 Internal Medicine, LewisGale Medical Center, Salem, USA; 3 Psychiatry, LewisGale Medical Center, Salem, USA

**Keywords:** stroke, pseudobulbar palsy, autonomic-voluntary dissociation, anterior opercular syndrome, foix-chavany-marie syndrome

## Abstract

Foix-Chavany-Marie syndrome (FCMS) is characterized by bilateral facio-glosso-pharyngo-masticatory paralysis of voluntary muscles due to bilateral infarction in the anterior opercular region of the brain. Here, we report a case of a 52-year-old female who presented with FCMS due to an acute left anterior opercular stroke in the setting of a chronic asymptomatic right opercular infarct and asymptomatic bilateral cerebellar infarcts. She also had a concurrent acute-on-chronic episode of congestive heart failure exacerbation. She made a significant recovery by the time of hospital discharge.

## Introduction

Foix-Chavany-Marie syndrome (FCMS), also known as anterior opercular syndrome, is a rare neurological condition that presents with voluntary facial, pharyngeal, lingual, and masticatory paralysis bilaterally [1]. Patients typically present with acute-onset facial weakness, dysphagia, and dysarthria or anarthria while maintaining the involuntary ability to yawn, smile, laugh, and cry [2,3]. This syndrome was first described by Magnus in 1837 and further defined by Foix, Chavany, and Marie in 1926 after whom the condition was later named [4]. FCMS is commonly caused by ischemic lesions [1-4]; however, it has also been reported in patients with cerebral infection [5-7], demyelinating disease [8], trauma [9], tumor [10], hydrocephalus [11], and osmotic demyelination syndrome [12]. There have been less than 150 cases of FCMS noted in the literature, and less than 40 cases of which have been caused by ischemic stroke; however, FCMS is being increasingly reported due to advances in computed tomography (CT) and magnetic resonance imaging (MRI) [3,4].

## Case presentation

A 52-year-old right-handed female presented to the emergency department after waking up in the morning and acutely not being able to open her jaw. She also reported having shortness of breath, dysphagia, tongue numbness, and anarthria for several hours. Her past medical history was significant for hypertension, hyperlipidemia, congestive heart failure (CHF) with reduced ejection fraction (20-25%), atrial fibrillation, aortic stenosis, type 2 diabetes mellitus, chronic obstructive pulmonary disease, gastroesophageal reflux disease, hypothyroidism, and prior stroke five years ago without any residual deficits. She reported being compliant with her home medications, which included apixaban, carvedilol, lisinopril, furosemide, atorvastatin, insulin, glipizide, and levothyroxine, among others.

On admission, she was afebrile, normotensive, tachycardic, and tachypneic on her home oxygen therapy requirement of 2 liters of oxygen via nasal cannula. On physical examination, she was awake, alert, oriented, and tearful. There was no active drooling. She was unable to form speech, but there was no receptive aphasia. She communicated by writing on a notepad and answered all questions appropriately. Auscultation of the lungs revealed diffuse rhonchi and labored respiratory effort.

On neurological examination, she had flaccid weakness of the lower face and was unable to smile, puff out her cheeks, protrude her tongue, or move her tongue from side to side. There was no deviation of her tongue. She had a marked reduction in the range of motion of her jaw, and her muscle strength was 5/5 in all extremities with normal deep tendon reflexes. Cerebellar testing was unremarkable as well.

Laboratory results on admission (Table 1) were significant for troponin level of 426 ng/L, B-type natriuretic peptide level of 4522 ng/L, red blood cell count of 5.06 x 106/uL, glucose level of 226 mg/dL, albumin level of 3.3 g/dL, and alkaline phosphatase level of 138 U/L.

**Table 1:** Laboratory results on admission.

Test	Result	Reference range	Units
Hemoglobin	12.8	11.4-15.5	g/dL
Hematocrit	41.3	37.0-47.0	%
Red blood cell count	5.06	3.80-5.00	x 10^6^/uL
White blood cell count	12.14	4.50-10.50	x 10^3^/uL
Platelet count	234	130-385	x 10^3^/uL
Sodium	136	135-145	mmol/L
Potassium	4.5	3.6-5.2	mmol/L
Chloride	107	100-108	mmol/L
Carbon dioxide	23	21-32	mmol/L
Glucose	226	74-106	mg/dL
Blood urea nitrogen	17	7-18	mg/dL
Creatinine	0.95	0.60-1.30	mg/dL
Total protein	7.5	6.4-8.2	mg/dL
Albumin	3.3	3.4-5.0	g/dL
Calcium	9.1	8.5-10.1	mg/dL
Total bilirubin	0.6	0.2-1.0	mg/dL
Aspartate aminotransferase	23	15-37	U/L
Alanine aminotransferase	15	13-61	U/L
Alkaline phosphatase	138	45-117	U/L
Creatine kinase	76	21-215	U/L
Troponin	426	<53.7	ng/L
B-type natriuretic peptide	4522	5-125	pg/mL

**Figure 1:**
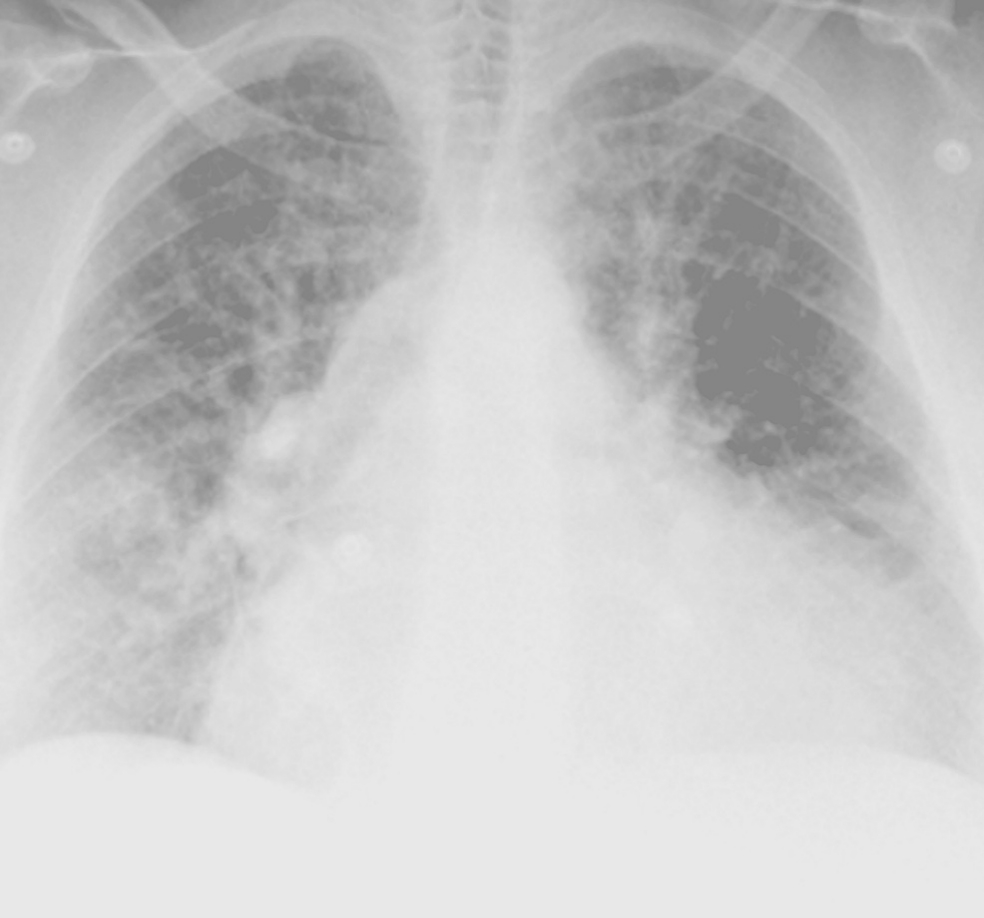
Chest X-ray showing widespread interstitial infiltrates and cardiomegaly due to pulmonary edema.

**Figure 2:**
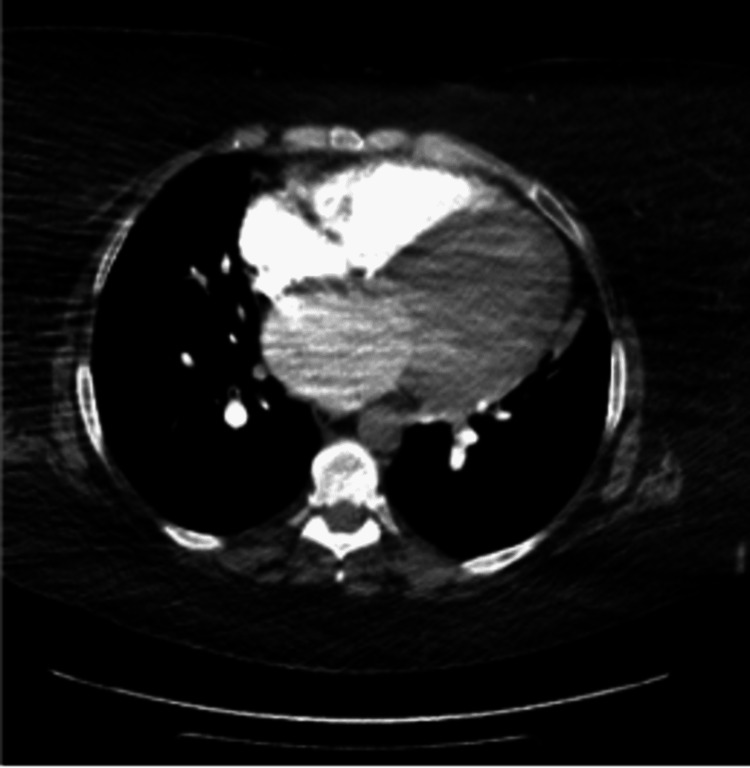
Computed tomography angiography of the chest showing interstitial edema, mild bibasilar atelectasis, and cardiomegaly.

An electrocardiogram showed sinus tachycardia with first-degree atrioventricular block and right axis deviation but no acute ischemic changes. Chest X-ray showed widespread interstitial infiltrates and cardiomegaly due to pulmonary edema (Figure 1). Computed tomography angiography of the chest showed interstitial edema, mild bibasilar atelectasis, and cardiomegaly (Figure 2). Echocardiography of the heart showed left ventricle dilation with severe diffuse hypokinesis, reduced systolic function, and ejection fraction of 15-20%. There was also moderate-to-severe aortic stenosis and moderate pulmonary hypertension with a pulmonary artery systolic pressure of 55 mmHg.

A modified barium swallow study showed silent aspiration of thin liquids. CT of the neck did not show any abnormalities. Carotid ultrasound showed patent (<50% stenosis) bilateral common and internal carotid arteries.

**Figure 3:**
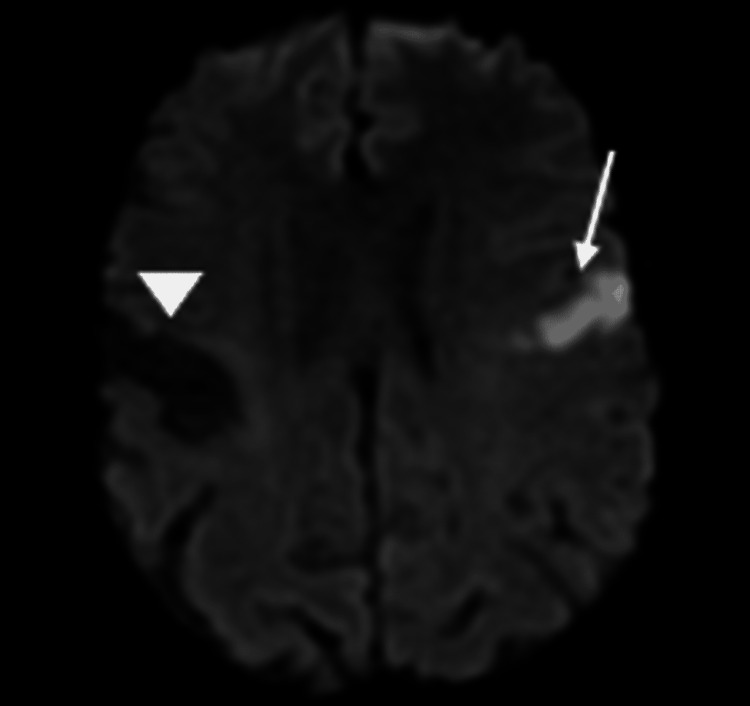
Diffusion-weighted MRI of the brain showing increased signal intensity in the region of the left frontal operculum (arrow), which indicates an acute stroke. Decreased signal intensity in the region of the right frontal operculum (arrowhead) is from a chronic infarct.

**Figure 4:**
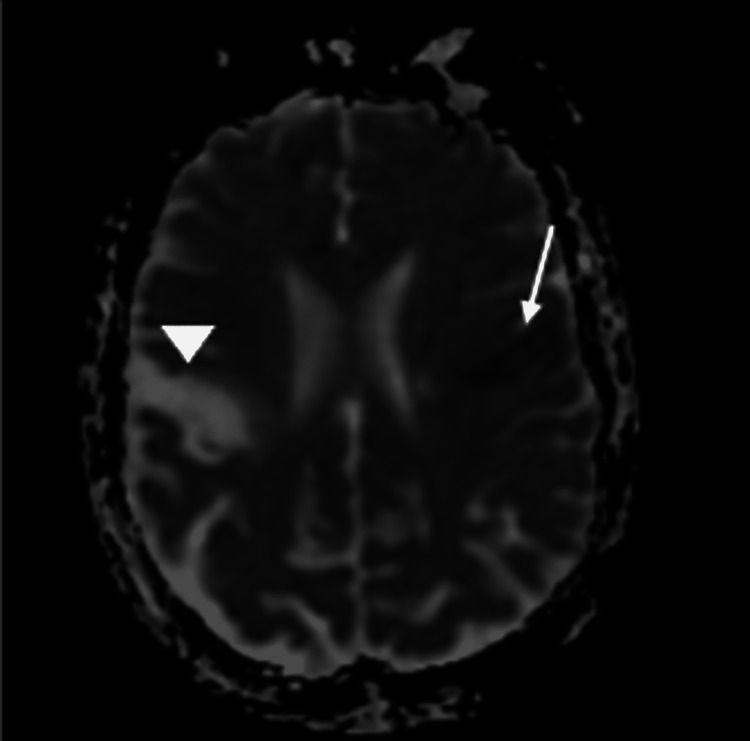
Apparent diffusion coefficient MRI of the brain showing decreased signal intensity in the region of the left frontal operculum (arrow), which confirms an acute left opercular stroke. Increased signal intensity in the region of the right frontal operculum (arrowhead) confirms a chronic infarct.

**Figure 5:**
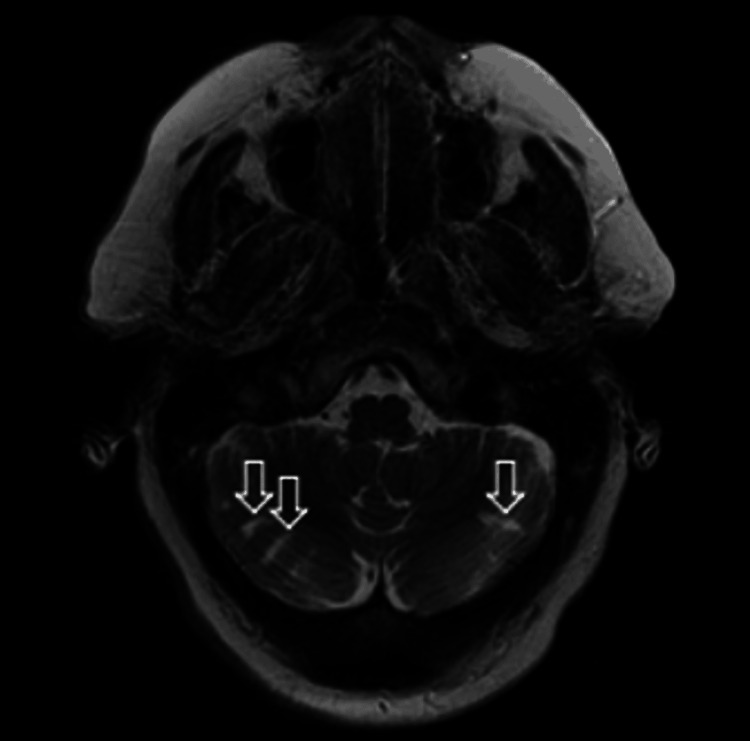
Apparent diffusion coefficient MRI of the brain showing increased signal intensity in the cerebellum bilaterally due to chronic infarcts (open arrows).

MRI of the brain delineated an acute left opercular infarct with a chronic right opercular infarct on diffusion-weighted imaging (Figure 3), which was confirmed with apparent diffusion coefficient imaging (Figure 4). Chronic bilateral cerebellar infarcts were also seen on apparent diffusion coefficient imaging (Figure 5). No hemorrhage or masses were noted, but there was evidence of mild cerebral atrophy and chronic small vessel ischemic disease.

Her home medication regimen was modified to adequately treat her current episode of CHF exacerbation with concurrent, type-2, non-ST-elevation myocardial infarction and acute hypoxic respiratory failure. Serial troponin levels were obtained until they trended downwards. She had presented to the emergency department beyond the time window for intravenous thrombolytic therapy. She was briefly anticoagulated with heparin, which was discontinued to prevent hemorrhagic conversion. Her antihypertensive medications were also held to optimize cerebral blood flow. Within 48 hours, she had noticeable improvement and was able to form speech with associated dysphonia. She was discharged from the hospital five days later to rehabilitation after significant improvement in speech, swallowing, and movement of her tongue, as well as optimal medical management of her acute-on-chronic CHF exacerbation.

## Discussion

FCMS results from vascular injury to the anterior operculum, which contains motor fibers for cranial nerves V, VII, IX, X, and XII [4]. The operculum comprises the cerebral cortex covering the insula, inferior frontal, precentral, postcentral, supramarginal, angular, and superior temporal gyri [13]. FCMS presents as voluntary paralysis of the facial, lingual, pharyngeal, and masticatory muscles bilaterally [4]. Preservation of reflexive and involuntary functions of the affected muscles is referred to as autonomic-voluntary dissociation, which is a hallmark of FCMS [4]. Autonomic-voluntary dissociation occurs due to alternative pathways connecting the amygdala and hypothalamus that allow for facial emotional expression and involuntary movements [2]. This phenomenon was consistent with our patient’s presentation because she had objective weakness of the lower face and deficits on neurological examination but intact emotional expression congruent with not being able to speak, namely, anxiety, fear, sadness, frustration, and tearfulness.

FCMS corresponds to the cortical subtype of pseudobulbar palsy and can be distinguished from the basal ganglia and brainstem subtypes by the lack of emotional incontinence, urinary dysfunction, and peripheral muscle tone abnormalities [14]. FCMS can be differentiated from other conditions, such as bulbar paralysis, cranial nerve palsies, myasthenia gravis, and botulism by intact extraocular movements, preserved brainstem reflexes, and the absence of lower motor neuron signs (atrophy and fasciculations) [13]. The symptom of trismus alone can be caused by a myriad of conditions, such as temporomandibular joint dysfunction, masseter spasm, tetanus, giant cell arteritis, malignancy, and odontogenic, tonsillar, or pharyngeal infection [15]. The constellation of symptoms of FCMS may also have a non-organic or psychiatric component in the differential diagnosis as well.

Although the exact mechanism by which FCMS occurs is unknown, it is hypothesized that the presence of asynchronous contralateral lesions serves as the catalyst for FCMS [1,13,14]. There have been rare reports of FCMS caused by unilateral opercular lesions, but there is a possibility that those cases might have had subtle contralateral lesions not visible on brain MRI [1,13,14]. Our patient’s brain MRI findings elucidated an acute left opercular infarct with a chronic right opercular infarct, which corroborates this hypothesis. Our patient suffered a stroke five years prior without having any residual deficits, which could explain the asymptomatic right opercular infarct seen on brain MRI. We believe that the sequential nature of the old right opercular lesion followed by the new left opercular lesion led to the manifestation of FCMS in our patient.

Furthermore, our patient had chronic asymptomatic cerebellar infarcts bilaterally. Torres-Perales et al. detailed a case report of FCMS with a unilateral opercular lesion and a chronic cerebellar lesion; they propounded that this association between the opercular and cerebellar infarcts is more than a simple coincidence [16]. Sá et al. also presented a case report of FCMS with a unilateral opercular lesion associated with a chronic asymptomatic contralateral cerebellar lesion [1]. As a result, our patient’s findings support the proposition put forth by Torres-Perales et al. since the cerebellum plays a substantial role in voluntary motor activity.

The majority of patients with FCMS due to bilateral opercular lesions have a poor prognosis with persistent deficits in chewing, swallowing, and speech functions [1,13]. Despite having asynchronous bilateral opercular lesions with concomitant acute-on-chronic CHF exacerbation, our patient made a significant recovery. The only residual symptom she had at the time of hospital discharge was mild dysphonia.

## Conclusions

Although FCMS is a rare neurological condition, the diagnosis should be considered in patients presenting with acute voluntary muscle loss of the face, tongue, and pharynx with autonomic-voluntary dissociation. Early recognition of these uncommon signs and symptoms of FCMS is essential to making the correct diagnosis for potential acute intervention, nutritional support, rehabilitation, and secondary prevention.
